# Refractory vasculitic ulcer of the toe in adolescent suffering from Systemic Lupus Erythematosus treated successfully with hyperbaric oxygen therapy

**DOI:** 10.1186/1824-7288-36-72

**Published:** 2010-10-31

**Authors:** Alma N Olivieri, Antonio Mellos, Carlo Duilio, Milena Di Meglio, Angela Mauro, Laura Perrone

**Affiliations:** 1 Department of Pediatrics, Second University of Naples, Via L. De Crecchio 2, 80138 Naples, Italy; 2 CEMSI - Institute of Underwater and Hyperbaric Medicine, Salerno, Italy

## Abstract

Skin ulcers are a dangerous and uncommon complication of vasculitis. We describe the case of a teenager suffering from Systemic Lupus Erythematosus with digital ulcer resistant to conventional therapy, treated successfully with Hyperbaric Oxygen Therapy. The application of hyperbaric oxygen, which is used for the treatment of ischemic ulcers, is an effective and safe therapeutic option in patients with ischemic vasculitic ulcers in combination with immunosuppressive drugs. Further studies are needed to evaluate its role as primary therapy for this group of patients.

## Introduction

Vasculitis is a heterogeneous group of diseases characterized by inflammatory processes of the blood vessel wall resulting in the alteration of blood flow and vascular damage. The vessels involved are of different sizes (arteries, arterioles, venules, capillaries). Resulting syndromes are the consequence of tissue ischemia, vascular damage and systemic inflammation. Most vasculitis is mediated by the vascular deposition of immune complexes (IC), abnormal cell-mediated immune responses, antibodies against endothelial cells or against lysosomal enzymes of neutrophils. Vasculitis may be primary or secondary to connective tissue and intestinal diseases, medications, tumors or athopy. Vasculitic skin ulcers are clinical manifestations of ischemic injury, and are usually treated medically by controlling the underlying disease with immunosuppressants and systemic vasodilators [1]. Hyperbaric oxygen therapy (HBOT) is used as primary or adjuvant therapy in various clinical conditions, including cutaneous vasculitic ulcers that are resistant to immunosuppressive therapy [2]. We present a case of refractory vasculitic ulcer responding to hyperbaric oxygen (HBO), which was used in combination with immunosuppressive therapy. We decided to treat the infected ulcer of the patient with HBOT not only because it improves the oxygenation of ischemic tissues and exerts beneficial effects on vascular inflammatory response by regulating the chemotaxis of leukocytes, but also because it facilitates the healing process of infected wounds promoting the deposition of collagen, angiogenesis, epithelialization and facilitating the oxygen-dependent killing by leukocytes.

## Case report

**Figure 1:**
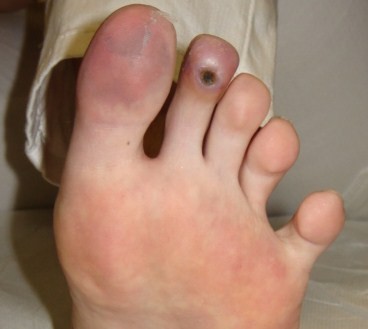
**Ulcer as it appears after 5 sessions of HBO**.

**Figure 2:**
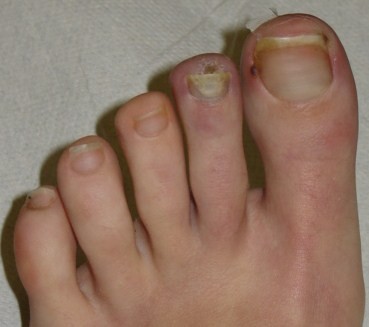
**Toes as they appear after 5 sessions of HBO**.

**Figure 3:**
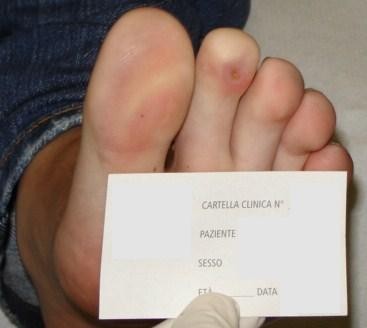
**Toe as it appears after 32 sessions of HBO therapy**.

In mid February 2008 a 14 year old girl came under our observation. She had been suffering for about 3 months from persistent fever (38 ° C), weight loss (9 kilos), headache and asthenia and was previously treated with several courses of antibiotics and prednisone. Family history shows that her paternal grandmother suffers from scleroderma, her paternal grandfather from rheumatoid arthritis, her maternal grandmother from autoimmune thrombocytopenia, and her mother from thyroiditis. At admission, she had a body weight of 45 kg (10^th ^-25^th ^percentile) and a height of 154 cm (10^th ^-25^th ^percentile). Physical examination revealed a rash on the face, livedo reticularis, acrocyanosis at the first and second toe of the left foot, arthritis in knees and ankles. Blood tests showed: anemia (hemoglobin 10.5 gr/dl), leukocitopenia (3300/μl) with lymphopenia (30%), normal platelet count (289000/μl), increased inflammatory indexes (erythrocyte sedimentation rate 53 mm/1 hour; C-reactive protein 1,44 mg/dl); increased serum IgG (2270 mg/dl), lengthening of prothrombin time (57.5 seconds), reduced serum iron (16 μg/dl) and reduced levels of C3 (70 mg/dl) and C4 (9 mg/dl). Urinalysis excluded renal involvement, and instrumental examinations (chest x-ray, abdominal ultrasound, echocardiography) ruled out the presence of serositis or other signs of disease. An eye examination, echocolordoppler of lower limbs and capillaroscopy were also negative. A diagnosis of Systemic Lupus Erythematosus (SLE) was considered and confirmed, according to the diagnostic criteria laid down by American College of Rheumatology, for the presence of antinuclear antibodies (1:1280, homogeneous appearance), anti-dsDNA (107 IU/ml; normal < 30), anti-Sm (129 AU/ml, normal <30), anti-Sm/RNP (120 AU/ml, normal <30), lupus anticoagulant (1.48; normal 0-1.3) and anticardiolipin antibodies (25 U/ml, normal up to 20). Her lupus anticoagulant and anticardiolipin antibodies were positive on one occasion, but subsequently negative with no other evidence to suggest anti-phospholipid syndrome. The patient was discharged with the following therapy: prednisone (60 mg daily); hydroxychloroquine (200 mg); anti-aggregating platelet therapy (acetyl salicylic acid); ranitidine; vitamin D3 and calcium carbonate. After two weeks of therapy there was an improvement in the symptoms with normalization of biochemical indices of inflammation but a worsening of peripheral cyanosis, with the appearance of an intensely painful and infected ischemic ulcer on the plantar surface of the second toe of the left foot. Echocolordoppler and capillaroscopy were again performed and were both negative. A local ulcer treatment was started with medications and cleansing performed by a consultant surgeon. The ulcer, oval in shape, evolved in a pejorative sense at a distance of 3 months after onset, reaching a maximum diameter of about 1 centimeter. For this reason, and because of the deepening of the lesion and the risk of osteo-tendinous impairment, it was decided to associate with the medical therapy a course of HBOT, with a protocol of 5 weekly sessions of 90 minutes at a pressure of 2.6 atmospheres absolute (ATA). Before starting the hyperbaric therapy, the ulcer appeared painful and oval shaped with regular margins. It was covered by an infected eschar and the surrounding skin was cold, thick and ranging from deep red to bluish in color. After a week of therapy, the cyanosis disappeared (figures 1 and 2), while the ulcer healed after 16 sessions. The HBO was well tolerated by the patient and 32 sessions were carried out in total without any side effects (figure 3). From the interruption of hyperbaric therapy, there were no relapses in the following months. After four weeks from the beginning of the immunosuppressive therapy, the daily dosage of prednisone was reduced by 5 mg every 15 days; and when it reached 20 mg/day, it was further reduced by 2.5 mg every four weeks until the current dose of 10 mg/day. The immunosuppressive therapy was well tolerated by the patient, blood tests were normal and there have been no new flares of the disease so far.

## Discussion

Vasculitic skin ulcers are a manifestation of muscular-vessel vasculitis, and are very painful and difficult to manage. They are usually treated by medical therapy with systemic anti-inflammatory drugs, steroids and immunosuppressants. For the treatment of refractory vasculitic skin ulcers the use of prednisone combined with other drugs such as methotrexate, azathioprine, mycophenolate mofetil, cyclophosphamide or hydroxychloroquine is recommended. Even the administration of intravenous immunoglobulin and plasmapheresis has been effective in several case reports [1]. Other promising therapeutic options for these patients are vasodilators such as antagonists of endothelin (Bosentan) and analogs of prostacyclin (Iloprost) [1,3-5]. Not always, as in our case, the ulcers heal with systemic treatment; for this reason and because of the beneficial effects on healing of infected vasculitic lesions, it was decided to treat this patient's refractory ulcer using the hyperbaric oxygen (HBO)[2,6,7]. A case of SLE complicated by vasculitic ulcers initially treated unsuccessfully with high doses of steroids and immunosuppressants (mycophenolate mofetil and cyclophosphamide) showing a significant improvement after only 2 doses of rituximab, 3 months of methotrexate and 1 month of HBOT has been recently described in a female adult [7]. Moreover, a recent study evaluated the effects of HBOT on 35 adult patients with refractory vasculitic skin ulcers that were not responding to immunosuppressive therapy. Seven of these 35 patients had SLE. The treatment included four weeks of HBO (5 weekly sessions of 90 minutes at 2 ATA). Satisfactory results were obtained: at the end of hyperbaric therapy 28 patients (80%) were completely cured, four (11.4%) only partially, and in three (8.6%) there was no improvement. Only one of these three patients who did not respond to the hyperbaric treatment had SLE. None of these 35 patients had side effects and all of them tolerated the hyperbaric therapy well [2]. In this study, authors used the University of Texas Wound Classification System to evaluate ulcer severity [8]. HBO is a treatment that consists of the administering of 100% pure oxygen in a pressurized chamber to an absolute atmosphere (ATA) greater than 1, leading to an increase in the amount of free oxygen in the plasma, creating a diffusion gradient that facilitates the transition of oxygen from the capillaries to the ischemic tissues. It is particularly effective against anaerobic bacteria facilitating the killing by leukocytes, and increasing the effectiveness of various antibiotics by acting in synergy. It also promotes the deposition of collagen, angiogenesis, epithelialization and exerts beneficial effects on vascular inflammatory response, reducing the adhesion of neutrophils in the microcirculation [9-11]. HBO exerts its anti-bacterial effect facilitating the oxygen-dependent peroxidase system by which leukocytes kill bacteria and increasing generation of oxygen free radicals which oxidize proteins and membrane lipids, damage deoxyribonucleic acid (DNA) and inhibit metabolic functions of bacteria. HBOT also improves the oxygen-dependent transport of certains antibiotics across bacterial cell walls, particularly aminoglycosides [9,10]. Several studies show that HBO increases the proliferation of fibroblasts and endothelial cells and it also induces the differentiation and migration of keratinocytes. Moreover, fibroblasts need oxygen tensions to deposit collagen properly and the production of collagen is proportional to oxygen tensions. The major enzymes (prolyl hydroxylase, lysil hydroxylase, lysil oxydase) involved in the post-translational steps of collagen synthesis require oxygen as a cofactor. In fact, oxygen is needed for lysine and proline hydroxylation, a step required for the collagen release from cells. Without oxygen, the underhydroxylated pro-alpha peptide chains fail to form a triple helix. Prolyl hydroxylase is required not only for hydroxyproline synthesis but is also essential for triple helix formation, while lysil hydroxylase and lysil oxidase allow for proper collagen cross-linking. It has been experimentally demonstrated that vascular endothelial growth factor (VEGF) increases its expression in both hypoxic and hyperoxic conditions, but angiogenesis proceeds more efficiently and it can only be maintained with sufficient oxygen tensions [11]. Oxygen stimulates reepithelization via reactive oxygen species (ROS), which are necessary for the function of growth factors, such as the epidermal growth factor (EGF) [11], and hyperoxia decreases edema in the periwound area through its vasoconstrictive action [9]. HBO acts on an important pathogenic mechanism because it reduces rolling and adhesion of polymorphonuclear cells in the microcirculation, affecting polymorphonuclear-endothelial cell adhesion via the modification of CD receptors, thus downregulating the functions of CD11/18 [2]. In fact, it is thought that the increased expression of vascular adhesion molecules have an important role in the pathogenesis of vasculitis secondary to connective tissue diseases [1]. Oxygen therapy is generally safe and well tolerated [2]. Side effects are generally mild and reversible, and rarely occur if the applied pressure is less than 3 ATA and if the duration of the sessions is limited to a maximum of 120 minutes [12]. Possible complications are given by barotraumatic lesions, oxygen toxicity, confinement anxiety, myopia, and cataracts [9-11]. Absolute contraindications for this treatment method are untreated pneumothorax and use of chemotherapy. Relative contraindications include known malignancies, pregnancy, implanted pacemakers, upper respiratory infections, chronic sinusitis, epilepsy, emphysema, hyperthermia, history of thoracic surgery, optic neuritis, otosclerosis, viral infections and congenital spherocytosis [10].

## Conclusion

We decided to start HBOT before adding other immunosuppressants to the basic therapy, because these drugs are sometimes ineffective and not free from side effects, and also because the patient, other than her vasculitic ulcer, had no signs of disease activity and her blood-chemical indices were normal. Our case demonstrates the usefulness and effectiveness of this treatment in healing infected ischemic lesions. Before starting the cycle of hyperbaric oxygen, the patient's ulcer was grade 1 (superficial wound with involvement of the dermis and skin, but without compromising joint, tendon or bone) and stage D (ischemic wound infection), according to the classification system of wounds of the University of Texas. At the end of the sessions, the lesion became Grade 0 (healed ulcer) and stage A (no signs of suffering disease or infection). The lack of serious side effects and relatively low cost makes this treatment beneficial for patients with ischemic vasculitic ulcers. Currently, case-control or retrospective studies on the effectiveness of HBOT in young patients with SLE complicated by vasculitic skin ulcers have not been listed until now. Considering the potential complications and the lack of large retrospective studies or case-control studies, further research is needed to determine its effectiveness and its role as primary therapy for vasculitic ulcers both in pediatric patients and adults.

## Consent

Written informed consent has been obtained from the patient and her relatives for the publication of this case report and 3 accompaying images. A copy of the written consent is available for review by the Editor in Chief of this journal.

## Competing interests

The authors declare that they have no competing interests.

## Authors' contributions

ANO took care of the patient during the hospitalization and follow-up, formulated diagnosis, wrote the manuscript and was responsible for its review. AM was involved in the clinical care and follow-up of the patient; he wrote the manuscript and was responsible for its review. He provided to translate and send the manuscript. CD was in charge of hyperbaric oxygen therapy for the patient, gave three pictures appearing on this article and provided all details concerning this hyperbaric oxygen treatment; he contributed to evaluating of the manuscript. MDM was involved in the clinical care and follow-up of the patient and participated in the design of the manuscript. AGM was involved in the follow-up of the patient and participated in the design of the manuscript. LP took care of the patient during the hospitalization, evaluated the manuscript and participated in its design. All authors read and approved the final manuscript.
